# On the Differential Diagnosis of Anxious from Nonanxious Major Depression by means of the Hamilton Scales

**DOI:** 10.1155/2013/294516

**Published:** 2013-10-27

**Authors:** George Konstantakopoulos, Vasilios G. Masdrakis, Manolis Markianos, Panagiotis Oulis

**Affiliations:** ^1^First Department of Psychiatry, Athens University Medical School, Eginition Hospital, 11528 Athens, Greece; ^2^Section of Cognitive Neuropsychiatry, Department of Psychosis Studies, Institute of Psychiatry, King's College London, London SE58AF, UK

## Abstract

*Objective.* Anxious major depressive disorder (A-MDD) is differentially diagnosed from nonanxious MDD (NA-MDD) as MDD with a cut-off score ≥7 on the HAM-D anxiety-somatization factor (ASF). We investigated whether additional HAM-D items discriminate A-MDD from NA-MDD. Moreover, we tested the validity of ASF criterion against HAM-A, gold standard of anxiety severity assessment. *Methods.* 164 consecutive female middle-aged inpatients, diagnosed as A-MDD (*n* = 92) or NA-MDD (*n* = 72) by the normative HAM-A score for moderate-to-severe anxiety (≥25), were compared regarding 17-item HAM-D scores. The validity of ASF ≥7 criterion was assessed by receiver-operating characteristics (ROC) analysis. *Results.* We found medium and large effect size differences between A-MDD and NA-MDD patients in only four out of the six ASF items, as well as in three further HAM-D items, namely, those of agitation, middle insomnia, and delayed insomnia. Furthermore, the ASF cut-off score ≥9 provided the optimal trade-off between sensitivity and specificity for the differential diagnosis between A-MDD and NA-MDD. *Conclusion.* Additional HAM-D items, beyond those of ASF, discriminate A-MDD from NA-MDD. The ASF ≥7 criterion inflates false positives. A cut-off point ≥9 provides the best trade-off between sensitivity and specificity of the ASF criterion, at least in female middle-aged inpatients.

## 1. Introduction

Approximately half of patients with major depressive disorder (MDD) exhibit severe anxiety, that is, “anxious MDD” (A-MDD) [1, 2]. Higher levels of concomitant anxiety in MDD have been associated with greater functional impairment and a more chronic course of illness [1–5]. However, the severity threshold of concurrent anxiety required for the diagnosis of A-MDD remains unspecified. Extant research adopts as a diagnostic criterion of A-MDD the cut-off point ≥7 on the 6-item anxiety/somatization factor (ASF) of the Hamilton Depression Rating Scale (HAM-D) [6], comprising the items of psychic and somatic anxiety, general somatic and gastrointestinal symptoms, hypochondriasis, and lack of insight [1, 2]. However, to our knowledge, it has never been investigated whether further HAM-D items might help discriminate A-MDD from nonanxious MDD (NA-MDD). Moreover, even the proponents of this criterion acknowledge as one of its major limitations the fact that HAM-D captures only a limited number of anxiety symptoms and thus its exclusive use carries a significant risk for patients' misclassification [7, 8]. More precisely, the ASF-score criterion has never been validated against other more specific and comprehensive anxiety measures [1, 2]. One such clinical gold-standard is the Hamilton Anxiety Rating Scale (HAM-A) [9]. Furthermore, extant studies have been carried out mostly in outpatients with MDD, including mild cases as attested by the low cut-off score of only ≥14 on the HAM-D for patient recruitment [1]. Thus, more severely ill MDD patients in need of hospitalization are systematically under-represented in their otherwise very large sample sizes. Finally, ASF is a composite factor of both anxious and somatic symptoms of depressive illness and somatic symptoms are far more prevalent in female than in male patients [10]. This study aims to start filling these knowledge gaps by using concurrently HAM-D and HAM-A Scales in order to first investigate whether further HAM-D items discriminate A-MDD from NA-MDD and second assess the diagnostic validity of the ASF criterion in an exclusively female inpatient setting.

## 2. Methods

### 2.1. Participants and Assessments

One hundred eighty-four consecutive female inpatients with DSM-IV diagnosis of MDD in relapse participated in the study. Patients were admitted to the Women Inpatient Unit of our Department. Ethics Committee's approval and patients' written informed consent were obtained. Diagnosis was confirmed through the Structural Clinical Interview for DSM-IV Axis I Disorders [11] and a thorough clinical and laboratory workup in order to exclude cases of secondary major depressive episode due to medical conditions. On admission patients were rated concurrently on both HAM-D (17 items) and HAM-A. On the basis of the HAM-A normative for moderate-to-severe concomitant anxiety cut-off score of ≥25, patients were distinguished in A-MDD (*n* = 92) or NA-MDD (*n* = 72) groups. Eight patients of each group satisfied the DSM-IV specifier “with psychotic features.” All patients were screened during recruitment for pharmacological studies and were drug-free for at least one week with the exception of low-dose benzodiazepines (up to the equivalent to 5 mg of diazepam daily).  Table 1 displays patients' clinical and demographic characteristics.

### 2.2. Statistical Analyses

*t*-test was used for the analysis of continuous variables and chi-square test for categorical ones. All tests were 2-tailed. The magnitude of differences among the groups was assessed by Cohen's d metric of effect size [12]. The validity of the ASF ≥7 diagnostic criterion of A-MDD was assessed through receiver-operating characteristics (ROC) analysis [13]. SPSS 17.0 was used for the statistical analysis of data.

## 3. Results

No statistically significant differences were detected between the two inpatient groups with respect to age, total duration of depressive illness, presence of psychotic features at index episode, or menopausal status (see Table 1). The A-MDD group had significantly higher scores than NA-MDD group in both HAM-D and HAM-A, as expected.

A-MDD patients scored higher than their NA-MDD counterparts on the HAM-D items “middle” and “delayed insomnia,” “agitation,” “somatic anxiety,” “general-somatic” and “gastrointestinal symptoms,” and “hypochondriasis” with effect sizes ranging from medium to large (see Table 2). By contrast, the effect sizes for the ASF items “psychic anxiety” (NA-MDD: 2.72 ± 0.67, A-MDD: 2.87 ± 0.82) and “insight” (NA-MDD: 0.17 ± 0.51, A-MDD: 0.30 ± 0.55) were very small (0.20 and 0.24, resp.).

Table 3 displays the results of the ROC analysis: with an ASF cut-off point ≥7, patients were classified as A-MDD with a high sensitivity (91.3%), however with a very low specificity (22.2%). Instead, the cut-off point of ≥9 provided a much better trade-off between sensitivity (78.3%) and specificity (66.7%).

## 4. Discussion

We investigated the validity of the ASF factor of HAM-D in the differential diagnosis of A-MDD from NA-MDD in 164 consecutive middle-aged female inpatients with DSM-IV MDD in recent relapse. Patients were subtyped as anxious versus nonanxious on the basis of the HAM-A normative cut-off score of 25 for moderate-to-severe concomitant anxiety. Only four out of the six ASF items discriminated robustly the two subgroups, namely, somatic anxiety, general somatic symptoms, gastrointestinal symptoms, and hypochondriasis. Moreover, further HAM-D items not included in the ASF, namely, agitation, middle insomnia, and delayed insomnia, discriminated A-MDD from NA-MDD female inpatients with medium-to-strong effect sizes. Finally, the usual cut-off point of 7 on the ASF was found unsatisfactory. Instead, a cut-off point of at least 9 proved more valid.

A-MDD patients scored significantly higher on only four out of six ASF items: somatic anxiety, hypochondriasis, and somatic symptoms—general and gastrointestinal—with effect sizes ranging from upper medium to strong (0.66–0.94). Moreover, they exhibited more severe middle and delayed insomnia, with effect sizes in the upper medium-range (0.59 and 0.67, resp.). These items, especially the second, capture traditional vegetative core features of “endogenous depression” or melancholia. They are not included in the ASF, though it has been proposed that they should be component parts, along with somatic anxiety, hypochondriasis, and somatic symptoms—general and gastrointestinal— of a “somatic anxiety/somatization” factor [14]. Our findings concur with this proposal. Patient groups did not differ in the ASF items “psychic anxiety” and “insight.” Both these negative findings seem plausible since psychic anxiety is a near-universal concomitant of both A-MDD and NA-MDD, whereas lack of insight helps discriminate psychotic from nonpsychotic MDD rather than A-MDD from NA-MDD.

The ASF is based on the HAM-D factor analysis of Cleary and Guy [15]. Other HAM-D factor analyses proposed an anxiety/agitation factor comprising agitation, somatic anxiety, and psychic anxiety [16]. Our finding that the item of “agitation” was the strongest discriminator of A-MDD from NA-MDD with an effect size of 1.00 lends support to this proposal and suggests the strong clinical affinity of A-MDD with the traditional clinical MDD subtype of “agitated depression.” Moreover, agitation, as a behavioural expression of patients' anxiety levels, is a far more reliable clinical indicator of A-MDD than psychic anxiety, the assessment of which relies largely on patients' verbal reports. Overall, our findings suggest that the item composition of the ASF of the HAM-D should be modified by the inclusion of new items (agitation, late insomnia, and middle insomnia) and the possible exclusion of others, especially of the item of “insight,” at least in inpatient settings.

Adopting the HAM-A ≥25 cut-off point for diagnosing A-MDD is clinically justified, since anxiety at lower levels is a regular concomitant of even NA-MDD. Accordingly, the diagnosis of A-MDD should require at least moderate-to-severe anxiety, that is, HAM-A total score ≥25 [9]. Using this cut-off point as gold-standard in the present study, we found that the commonly accepted ASF criterion had high sensitivity (91.3%), but also very low specificity (22.2%). Instead, setting the ASF cut-off point at ≥9 provided the best trade-off between sensitivity and specificity (78.3% and 66.7%, resp.) with a small decrease of sensitivity (13%), yet with a threefold increase in specificity. Thus, the usual ASF criterion proved over-permissive, inflating spuriously false positive rates, at least in inpatient settings.

This finding has also important therapeutic implications. More precisely, recent research has shown that A-MDD patients as diagnosed by the usual ASF cut-off score ≥7 with a mean HAM-D score of 20.5 ± 4.2 do not respond differentially to a selective serotonin reuptake inhibitor (SSRI), namely, escitalopram, versus placebo. Such differential response was seen only in patients with a score of ≥26 on the 17-item HAM-D or ≥30 on the Montgomery-Asberg Depression Rating Scale. Likewise, only among the severely depressed patients, A-MDD predicted poorer response to SSRI treatment than NA-MDD [8]. Although the authors did not provide data on ASF scores of patients with severe A-MDD, from their total HAM-D score ≥26, we can plausibly surmise that their mean ASF score was above the cut-off point of  ≥7. Of note, the mean score on the HAM-D in the A-MDD group of our inpatient sample was close to this score (29.9 ± 5.3). Although beyond the scope of the present study, the differential diagnosis of A-MDD from NA-MDD may also provide clues to their differential aetiology. More precisely, in a recent large 10-year prospective community and family study comorbid MDD-GAD was associated with higher harm avoidance and a family history with a higher range of disorders, including mania. In contrast, MDD without comorbid GAD was associated with fewer and less severe depressive symptoms and higher odds for a family history with depression alone [17].

Limitations of the current study include its exclusively middle-aged female composition. However, anxious and somatic symptoms are far more prevalent in female than in male depressive patients [10]. At any rate, further research in adequate samples of larger age groups of both sexes is warranted.

In sum, additional HAM-D items, beyond those of ASF, discriminate A-MDD from NA-MDD. The ASF ≥7 criterion inflates false positives, whereas a cut-off point ≥9 provides the best trade-off between sensitivity and specificity of the ASF criterion, at least in female middle-aged inpatients.

## Figures and Tables

**Table 1:** Demographic and clinical characteristics of the sample.

	A-MDD (*n* = 92)		NA-MDD (*n* = 72)		*t*	*p*
	Mean	SD	Mean	SD		
Age	52.5	10.3	54.7	13.4	1.19	0.236
Duration of illness	12.1	13.5	14.8	10.2	1.41	0.160
HAM-D	29.9	5.3	24.8	5.7	5.92	<0.001
HAM-A	30.6	4.8	18.8	3.5	17.53	<0.001

	*n*	%	*n*	%	*χ* ^2^	

Menopausal	36	39.1	30	41.7	0.11	0.742
Psychotic features	8	8.7	8	11.1	0.27	0.605

A-MDD: anxious major depressive disorder; NA-MDD: nonanxious major depressive disorder; HAM-D: Hamilton Rating Scale for Depression; HAM-A: Hamilton Rating Scale for Anxiety.

**Table 2:** Medium and large effect size differences between A-MDD and NA-MDD groups on the 17-item HAM-D.

	A-MDD		NA-MDD		Effect size
	Mean	SD	Mean	SD	Cohen's *d*
Middle insomnia	1.3	0.9	0.8	0.8	0.59
Delayed insomnia	1.3	0.9	0.7	0.9	0.67
Agitation	0.9	0.5	0.4	0.5	1.00
Anxiety: somatic	2.6	0.8	1.8	0.9	0.94
Somatic symptoms: gastrointestinal	1.6	0.5	1.2	0.7	0.66
Somatic symptoms: general	2.0	0.0	1.8	0.4	0.71
Hypochondriasis	1.0	0.9	0.4	0.7	0.74

A-MDD: anxious major depressive disorder; NA-MDD: nonanxious major depressive disorder; HAM-D: Hamilton Rating Scale for Depression.

**Table 3:** Results of ROC analysis with HAM-A total score ≥25 as gold standard and ASF score as test variable.

ASF score	Sensitivity	Specificity
≥6	0.957	0.056
≥7	0.913	0.222
≥8	0.870	0.333
≥**9**	**0.783**	**0.667**
≥10	0.652	0.778

HAM-A: Hamilton Rating Scale for Anxiety; ASF: anxiety-somatization factor of Hamilton Rating Scale for Depression.
